# United or Divided? A Polarized Society’s Response to War

**DOI:** 10.1093/poq/nfag014

**Published:** 2026-04-03

**Authors:** Yuval Feinstein, Geffen Ben-David

**Affiliations:** Associate Professor, Department of Sociology, Vice Dean of Research, Faculty of Social Sciences, University of Haifa, Haifa, Israel; PhD Candidate, Department of Sociology, University of Haifa, Haifa, Israel

## Abstract

Recent global crises have renewed interest in the rally-’round-the-flag phenomenon of public opinion. At the same time, many studies have focused on deepening political polarization across countries. This study synthesizes these two lines of research by exploring how public opinion in a deeply polarized society responds to a security crisis that, in a less polarized context, would likely have led most citizens to close ranks behind a government that declared war on national enemies. We analyzed original panel data collected in Israel before and after the October 7 Hamas attack and during the subsequent Israeli military operation in Gaza. The findings reveal a split pattern: while the vast majority of Jewish Israelis supported the war and trusted the security forces, trust in the government and prime minister remained low. The analysis further identifies two distinct sets of mechanisms of attitude. Support for the war and trust in the security forces were associated with threat perceptions and anger about the enemy’s actions. In contrast, trust or mistrust in the government and prime minister hinged on whether respondents attributed blame for the crisis to the government or to the oppositional protest movement, an assessment tied to their preexisting views on the government’s controversial “judicial reform” initiative. These results suggest that extreme political polarization can prevent the emergence of a unified rally behind governments during severe security crises, mainly when internal strife produces contested views about the government’s responsibility for the crisis.

## Introduction

Over half a century ago, John Mueller (1970, 1973) borrowed the phrase “rally ’round the flag” from a US Civil War poem to describe sharp increases in public approval ratings of sitting US presidents. Interest in the rally phenomenon surged after the September 11 attacks (e.g., Hetherington and Nelson 2003; Perrin and Smolek 2009; Lambert et al. 2010) and was renewed during the COVID-19 pandemic (Baekgaard et al. 2020; Esaiasson et al. 2020; Goldfinch et al. 2021; Hamanaka 2021; Newton 2020; Yam et al. 2020) and the Russia-Ukraine war (Kulyk 2016; Theiler 2018; Erlich and Garner 2021; Bussmann and Iost 2024; Kizilova and Norris 2024). During the same period, ample studies have examined political polarization in many countries (for a review, see Bonikowski, Feinstein, and Bock 2021). This study synthesizes these two lines of research by exploring how a polarized society responds to a war that, in a less polarized context, would likely prompt most citizens to close ranks behind the government.

Early writing on the rally phenomenon did not consider the level of political unity in society as a potentially critical condition for its emergence. Mueller (1970, 22) expected a “let’s get behind the president” mood to emerge in response to any major international conflict directly involving the United States and its president. Earlier, Polsby (1964, 25) similarly observed that “invariably, the popular response to a president during an international crisis is favorable, regardless of the wisdom of the policies he pursues.” However, Waltz (1967) hinted that the emergence of a rally might depend on internal divisions, arguing that “an international event ordinarily does not disturb the nation unless it has first obsessed the government. In the face of such an event, the people rally behind their chief executive, *as one would expect them to do in any cohesive country*” (272; italics added). While the latter part of Waltz’s statement assumes cohesion in US society, it also implies that low internal cohesion may lead to weaker or absent rally effects.

While most research on the rally-’round-the-flag phenomenon over the past half century has focused primarily on the United States (for a review, see Feinstein 2022: chap. 2), studies have also documented rally periods in other countries (Norpoth 1987; Lanoue and Headrick 1998; Lai and Reiter 2005; Yudina 2015; Georgarakis and Sauger 2017; Feinstein 2018; Hale 2018; Theiler 2018; Bussmann and Iost 2024; Kizilova and Norris 2024; Chen 2025). These studies consistently identify security crises and wars as the most likely contexts for rally effects. Further, similar to the United States (Feinstein 2022), widespread feelings of national humiliation in response to enemy actions have often catalyzed rallying behind governments’ expected or ongoing military responses in other countries as well (Georgarakis and Sauger 2017; Sharafutdinova 2022). However, existing research has overlooked political polarization as a factor that may constrain rally effects, even during security crises involving widespread collective humiliation.

To examine how a polarized society reacts to a security crisis and war, we analyzed original survey data collected in Israel before and after the October 7, 2023, Hamas attack on civilian localities and military installations along the Gaza-Israel border and during Israel’s subsequent military operation in Gaza. The findings reveal a split pattern: while a majority supported the military action and trusted the military, the public remained divided in their views on the government and the prime minister (PM). The following sections develop a theoretical framework to explain these patterns. We first review the literature on political polarization, highlighting insights relevant to our case, then focus on how polarization shapes public reactions to external shocks. Next, we describe the characteristics of the October 7 attack and subsequent war, explaining why, under lesser polarization, they would likely have resulted in a significant rally behind the government. We then characterize the intensifying polarization in Israel in the months leading up to the October 7 attack and present a split attitudes model, which we subsequently test using survey data.

### The Growing Scholarly Interest in Political Polarization

Over the past decade, research on public opinion has increasingly focused on political polarization, often linking it to threats to democracy and the rise of radical-right authoritarian leaders (Mudde and Rovira Kaltwasser 2018; Silva 2018; Bischof and Wagner 2019; Hughes 2020; Axelrod, Daymude, and Forrest 2021; Bonikowski, Feinstein, and Bock 2021; Bonikowski, Luo, and Stuhler 2022; Dalton and Berning 2022; Elgenius and Rydgren 2022; Kotwas and Kubik 2022; Štefančík and Stradiotová 2022). However, the extant research has paid relatively little attention to how political polarization affects public reactions to external shocks, particularly security crises and wars. Instead, most studies focus on three aspects of polarization, all of which are essential background for interpreting the empirical findings of the present study.

The first aspect is a country’s polarization level, often assessed relative to other countries or earlier periods (Fiorina and Abrams 2008; Abramowitz 2010; Gidron, Adams, and Horne 2020). Below, we describe how recent events in Israel—the government’s attempt to implement a judicial reform, which triggered the largest protest movement in the country’s history (Porat 2023; Roznai and Cohen 2023)—intensified polarization by opening a rift between those who saw Israeli democracy as threatened and those who viewed the reform as strengthening it. This divide not only deepened the existing cleavage between supporters of right-wing parties and supporters of left-wing and center parties, but also fractured the right and contributed to party realignment.

A second aspect is the multifaceted nature of political polarization. Studies have identified two main dimensions of polarization: policy disagreements and “affective polarization” (i.e., antagonism between members of rival parties or ideological camps) (e.g., Mason 2015, 2018; Lelkes 2016; Iyengar et al. 2019; Wilson, Parker, and Feinberg 2020; Van Der Veen 2021). Other studies have distinguished between polarization among political elites and the general public (Green et al. 2020) and examined the relationship between rising public polarization and increased elite polarization (Garrett and Bankert 2020). Several studies have examined subjective aspects of polarization, including people’s beliefs about the level of polarization in their society, where they place themselves on the sociopolitical map, and how these assessments influence individual attitudes and, in aggregate, overall polarization (Baldassarri and Bearman 2007; Lelkes 2016; Levendusky and Malhotra 2016; Wilson, Parker, and Feinberg 2020; Kwon and Martin 2023).

In recent years, polarization in Israel has been high on multiple dimensions but intensified since early 2023, when internal strife centered on the government’s judicial reform initiative. The initiative triggered a confrontation between subsets of the Jewish majority who hold different national beliefs, embrace competing versions of Zionism, and hold contrasting visions for the country’s character and contract with its citizens (Feinstein 2023). The debate evolved into a passionate struggle between supporters of the reform, primarily aligned with right-wing and ultra-Orthodox parties, and opponents that included not only individuals from the political left and center, but also many from the right—including many people who voted for Likud in the most recent election (Porat 2023; see the Supplementary Material). As a result, on the eve of the October 7 attack, the majority of Israelis felt they were living in a highly polarized society (Gidron, Sheffer, and Mor 2022; Silver and Austin 2023).

The third aspect of polarization concerns the conditions and mechanisms driving its increase. Studies have shown that political elites, populist leaders, social media influencers, and algorithms create echo chambers that fuel fear and hatred of ethnic, racial, religious, or ideological “others” (Bail et al. 2018; Iyengar et al. 2019; Cinelli et al. 2021; Kawakatsu et al. 2021; Tokita, Guess, and Tarnita 2021; Gemici 2025; Nai and Maier 2023). People self-select into echo chambers and react negatively to opposing views, reinforcing a feedback loop between polarized media and a polarized public (Wilson, Parker, and Feinberg 2020; Prinz 2021; Zheng et al. 2024). During the research period, public polarization in Israel was coproduced by the operation of printed and electronic news outlets with an explicit right-wing ideological bent, the toxic conversation on social media during the judicial reform attempt and the protests against it, and the divisive discourse of the political elite (Nets-Zehngut 2023).

### External Shocks’ Impacts on Polarized Publics

Relatively scant scholarly attention has been paid to the effects of external shocks on polarized societies. However, some studies on public reactions to the COVID-19 pandemic have found that preexisting high political polarization has tempered the emergence of rally effects on public trust in governments and coping efforts (Green et al. 2020; Cardenal et al. 2021). Similarly, Macy et al. (2021) and Axelrod et al. (2021), who conducted experimental studies simulating various types of external shocks (i.e., economic crises, war, and a pandemic), found that, overall, these shocks did not derail polarization processes. Maxey (2022) further demonstrated through a series of survey experiments that affective polarization in the United States diminishes national unity in support of military actions, particularly in counterterrorism operations, but has a smaller effect on more widely supported humanitarian missions.

Myrick’s (2021) extensive analysis of public opinion polls in the United States over seven decades has led to a similar conclusion, showing that even when external threats reduce internal polarization and increase public approval ratings for the sitting president, these effects are typically short-lived, quickly overridden by polarizing domestic issues and electoral competitions. However, Myrick acknowledges (p. 937) that “high-threat” crises can lead to more substantial and longer-lasting reductions in polarization—as also highlighted by studies focusing on relatively rare but substantial rally periods (e.g., Parker 1995; Hetherington and Nelson 2003; Kam and Ramos 2008). Schwartz and Tierney (2025) further demonstrated through experiments that polarization reduction is more pronounced when an external threat is *vivid* and when there is broad agreement among policymakers about the nature of the threat and the appropriate response. Tama (2024) suggests that, more often than not, even when external threats elicit bipartisan policy responses, these do not reflect broader consensus among political elites or the public, while Feinstein (2016) shows that extreme events can produce significant rally effects in the public despite discordant elite discourse.

Whereas previous studies have offered initial clues about how polarized publics respond to security crises and wars, these clues are preliminary because they rely on experimental data with limited external validity or on surveys from events likely too minor to move people beyond their usual attitudes. As a result, they are insufficient for testing hypotheses about how polarized societies react to extreme security crises and wars. In contrast, we analyze data collected before and after a series of events that include all the characteristics identified by the literature as jointly sufficient to produce a sweeping rally-’round-the-flag effect. Furthermore, while previous studies have focused on long-standing ideological divisions, policy disagreements, and animosity toward political opponents—all of which have been characteristics of Israeli politics for years (Amitai, Gidron, and Yair 2023)—we identify an additional axis of polarization that arose in 2023 around the government’s judicial reform. This new divide cut across existing divisions and significantly shaped public responses to the October 7 attack and the ensuing war.

### Perfect Conditions for a Rally Following the October 7 Attack

According to Mueller’s (1970, 1973) seminal characterization, a rally requires an event that is international, dramatic, and sharply focused, with direct involvement of the country and its leader. Feinstein (2022) refined the conditions for rallies during security crises, identifying key factors: substantial military action against a widely hated enemy seen as the aggressor, widespread national humiliation, and leadership that employs “nation-affirming rhetoric” (Hutcheson et al. 2004, 28), promising to reclaim national honor and earn the respect of other nations through military action.

In the summer of 2014, a large military operation of the Israel Defense Forces (IDF) in Gaza following a series of rocket attacks by Hamas and the kidnap and murder of three Jewish settlers in the West Bank evoked a rally-’round-the-flag effect among the Jewish majority of Israel, most of whom supported the operation in Gaza and PM Netanyahu, whose approval ratings doubled and reached about two-thirds (Feinstein 2018). Nine years later, the security crisis and war that began on October 7, 2023, were much more intense and, therefore, could reasonably be expected to produce a sweeping rally in Israeli public opinion. The surprise attack by Hamas, which initially claimed the lives of 1,145 Israeli citizens (most of them civilians) and guest workers, destroyed many private homes, and abducted 251 people who were held hostage in Gaza, evoked the most intense feelings of threat since the 1973 war, collective humiliation, and desire to retaliate that cut across the political and ideological divide among Jewish Israelis, who frequently compared the situation to the Nazis’ crimes in the Holocaust.

Nearly all political leaders, except representatives of Arab parties, united behind calls for decisive military action in Gaza. The analogy to the Jewish suffering and struggle under Nazi Germany was applied by leaders from both coalition and opposition parties. In his speech in a special commencement of the Knesset to swear in an emergency government, PM Netanyahu said: “There are atrocities reminiscent of the days of the Holocaust. But we are not in the Warsaw Ghetto; we are deeply rooted in the homeland, the land of Israel. However, the lessons of the Warsaw Ghetto, 80 years ago, are etched into our national memory, in the face of the tyrant who sought to destroy, kill, and annihilate the Jewish people” (Eichner 2023 [authors’ translation]). In that speech, Netanyahu sought to mobilize public support for the war by fueling rage and the desire to react forcefully to Hamas’s attack. He stressed, “This is truly a war over our home. And it must end with one thing: complete victory and the crushing and elimination of Hamas.”

Past research has shown that anger is a primary emotion that, in response to terror attacks, drives individuals to support military action (Skitka et al. 2006; Huddy, Feldman, and Cassese 2007; Wayne 2023); that anger is often tied to concerns about the security and symbolic value of the nation rather than personal safety (Huddy et al. 2005; Huddy, Feldman, and Cassese 2007); and that during rally periods, anger-induced support for military operations is intertwined with increased public approval of the leadership that ordered the response (Lambert et al. 2010). Therefore, we may hypothesize that, in the wake of the October 7 attack, heightened anger—related to a perceived attack on their nation—would lead Israelis to unite not only behind military action but also behind the government and the prime minister.

To summarize, in the period following the October 7 attack, all conditions for a rally-’round-the-flag effect seemed to have been present in Israel: widespread feelings of humiliation and rage, a massive military operation that marked the target around Hamas’s personnel and installations in Gaza (but also claimed the lives of tens of thousands of civilians), and rhetoric by the political leadership that propagated the feelings of humiliation, anger, and desire for a forceful reaction.

However, experimental results suggest that extreme events can unite a divided public only if polarization has not already surpassed a “tipping point” and becomes irreversible (Axelrod, Daymude, and Forrest 2021; Macy et al. 2021). The extreme security crisis central to this study was preceded by heightened political polarization in Israel, fueled by the government’s judicial reform initiative and the massive protests it provoked (Porat 2023; Segev 2024; Simunovic, Dorfman, and Katzir 2024). Therefore, a counterhypothesis to the rally effect suggests that polarization in Israel had already surpassed the tipping point beyond which uniting behind the national leadership becomes impossible.

### The Extreme Polarization in Israel in the Months Before the Security Crisis

The October 7 attack and the subsequent IDF operation in Gaza found Israeli society polarized on two distinct but increasingly overlapping levels: policy issues and loyalty to political parties. Since early 2023, public debate has centered on the government’s judicial reform, which critics have labeled a “regime coup” (Feinstein 2025). Israel’s 37th government, sworn in January 2023, proposed several changes to shift judicial power toward the executive branch (Feinstein 2025; Roznai, Dixon, and Landau 2023). One of the main changes was limiting the Supreme Court’s authority to overrule government policies and parliamentary legislation. The legislation initiatives evoked the most extensive and prolonged protest in the country’s history.

The reform initiative and protest have become an increasingly polarizing issue. Our data reveal two key aspects of the intensification of political polarization in Israel (see the Supplementary Material). First, concerns about the future of democracy—prompted by the judicial reform—have emerged as a new axis of division, further separating the left and center from the right. Second, the judicial reform initiative has fractured the political right, prompting partisan realignment. For example, between May 2022 and April 2023, approximately one-third of those who voted for Likud in the November 2022 election withdrew their support for the party. Most of them expressed concerns about the future of Israeli democracy and either shifted to centrist parties opposing the judicial reform or reported that no political party currently represented them. By October 2023, partisan alignment around the judicial reform had largely solidified, and it remained despite the security crisis and war.

Amid the heated debate over the judicial reform initiative, some public figures expressed concerns about the potential impact of the political turmoil on national security. For example, on July 2, 2023, Knesset member and former IDF chief of staff Gadi Eisenkot expressed his concern that enemies’ interpretation of Israel’s polarization as a sign of weakness would motivate them to attack Israel (Eisenkot 2023). Opposition leader Yair Lapid has issued similar warnings (Lapid 2023). We mention these expressions of concern, which opinion polls found to be widely shared among Israelis before the October 7 attack (Israel Democracy Institute 2023), because, as we later show, after the attack, individuals who believed the judicial reform had contributed to the security crisis were more likely to disapprove of the prime minister and the government. In contrast, those who attributed the crisis to the anti-legislation protests, which they saw as having projected weakness, tended to evaluate the prime minister and the government more favorably.

Despite the divisive atmosphere, the October 7 attack has led to unprecedented mobilization of the civil society in Israel. The volunteer rate for reserved duty was extremely high (Amit 2023). Grassroots activists, including new associations, provided for the needs of victims of the terror attack and displaced families from villages and towns near the borders with Gaza and Lebanon, sent gear to soldiers, helped farmers to extract produce, and donated blood.

Considering the characteristics of the October 7 attack, Israel’s military reaction, and the rhetoric of the political leadership, we may expect the mobilization of civil society to have emerged in tandem with a sweeping rally. However, given previous studies on how polarized societies respond to external shocks, and preliminary findings suggesting that the October 7 attack did not significantly alter—and may have even slightly deepened—Israel’s political divide (Gidron 2024; Yair 2025), it is plausible that the hyper-polarization characterizing Israeli society since early 2023 shaped public reactions to the security crisis at the end of the year.

Specifically, we hypothesize that the anger-ex-threat mechanism identified in prior research on wartime public opinion (Huddy, Feldman, and Cassese 2007; Lambert, Schott, and Scherer 2011; Feinstein 2020) would primarily shape attitudes toward the war but not necessarily toward the government or its leader. Individuals who perceive an external threat may support a forceful response and continue to trust the military to carry it out, even if they see it as partly responsible, when its failures are viewed as operational and correctable. In contrast, a government that had already lost public trust before a security crisis due to policies seen as contrary to the national interest, may struggle to regain credibility, especially when large segments view it not just as operationally flawed but as contributing to the crisis through those policies. Our analysis examines whether, in the case of the public reaction in Israel to the October 7 attack, attributions of responsibility for the crisis were linked to preexisting views on the government’s judicial reform initiative and whether these assessments, in turn, were associated with distrust of the government during the war in Gaza. To address endogeneity concerns, the analysis relies on views about the judicial reform measured in March–April 2023, during the height of opposition to the reform and months before the security crisis and war.

**Figure 1.**
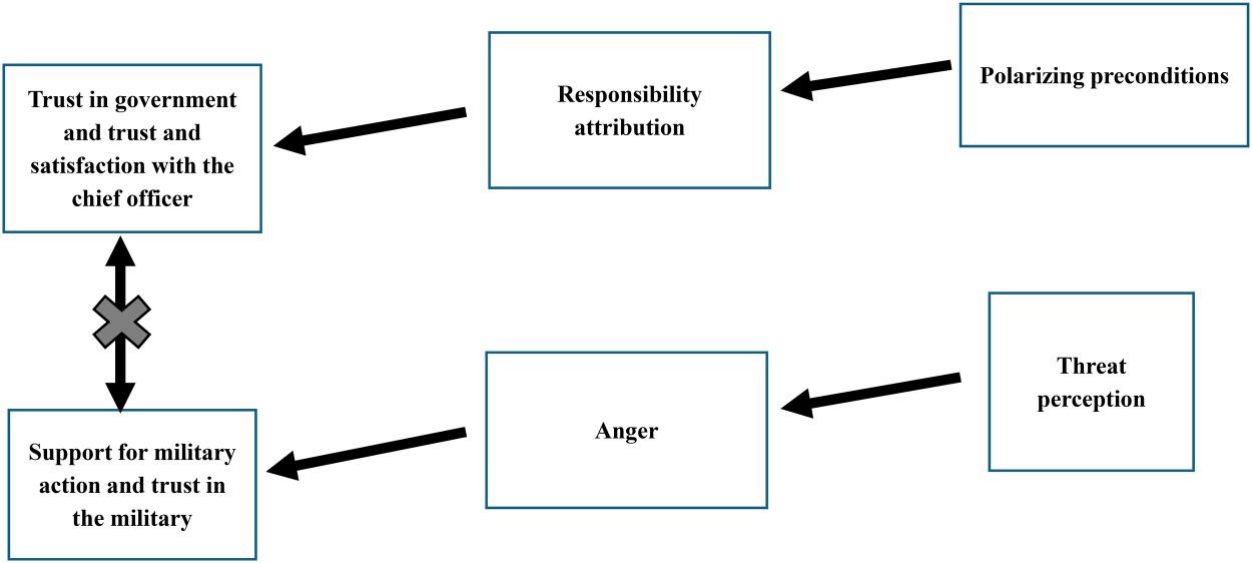
A split attitudes model.

Figure 1 splits public opinion into two sets of outcomes, each with its unique set of correlated variables.^1^ Note that the model includes an additional expectation: the X shape on the left indicates no association between attitudes about the war and those about the government and prime minister, which is another critical difference of the hypothesized split reaction from the sweeping rally effect—where people’s passionate support for military action tends to coincide with a rally behind the leaders who ordered and commanded it (Feinstein 2020).

Our hypothesized model proposes that individuals who support military action are also likely to trust the military. We expect that attitudes toward the military will diverge from attitudes toward the government and its leadership for two complementary reasons. First, as in many other countries, the military in Israel is regarded as a national institution. Consequently, citizens are unlikely to perceive the military as a political rival, and public attitudes toward it tend to be more cohesive than toward the governing political parties. Second, the IDF has long been central to Israeli society—not only as a military force but also as a pillar of nation building and civic identity. Since Israel’s foundation, the IDF has played a pivotal role in state building and social integration, deeply embedding militaristic values in society (Kimmerling 1993; Ben-Eliezer 1998). Meanwhile, military service has served as a mechanism for granting differential civic rights (Shafir and Peled 2002) and facilitating social mobility (Levy 2007). These characteristics help explain the high levels of trust in the IDF, which remains a core institution shaping Israel’s civic and societal landscape (Sheffer and Barak 2010; Feinstein and Ben-Eliezer 2019; Levy 2024).

In the months leading up to the October 7 attack, the embeddedness of military service in civic life was evident in the prominence of groups like Achim Laneshek (Brothers in Arms) and Lochamei 73 (Warriors of the 1973 War), both of which played a central role in the protest movement against the judicial reform. Alongside threats by reserve fighter pilots to suspend their service, these groups articulated not only a demand to safeguard the independence and authority of the Supreme Court but also a call for the government to compel ultra-Orthodox Jewish Israelis to share the burden of military service—an obligation that these groups, along with the majority of participants in the protest movement who subscribe to an ethno-republican model of citizenship (Peled 1992), regarded as the highest civic virtue (Feinstein 2023). This call for compulsory military service also resurfaced in public discourse during the Gaza war, which saw the extensive deployment of reserve soldiers. Moreover, following the October 7 attack, Achim Laneshek coordinated civil society’s efforts to provide logistical support for troops and assist farmers along the Gaza border in coping with war-related damage to their farms and produce—activities that illustrate how the boundaries between civilian and military spheres become especially blurred in Israel during wartime.

### Overview of the Two Competing Sets of Hypotheses

Our data analysis examines two competing scenarios, both of which include support for military action, which is associated with perceptions of security threats and anger toward the enemy. Both scenarios also maintain the high levels of trust in the military that characterize Israeli society during more peaceful periods; thus, these high levels alone do not constitute a rally-’round-the-flag effect. However, only one scenario includes an increase in favorable evaluations of the government and its leader. In this “rally” scenario, public trust in the government and prime minister, as well as satisfaction with the prime minister’s performance, rise substantially compared to precrisis levels.^2^ In this scenario, support for military action is positively associated not only with trust in the military but also with trust in the government and prime minister, and all these variables are positively correlated with perceived threats to national security and anger toward the enemy. In the second scenario, “polarization,” while trust in the military remains strong, trust in the government and the prime minister, along with satisfaction with the prime minister’s performance, do not increase substantially. Moreover, in this scenario, perceived threat and anger do not increase people’s likelihood of holding positive assessments of the government and the prime minister. Instead, these assessments depend on individuals’ prior views on divisive issues—in our case, the judicial reform initiative—and whether they blame the government for the security crisis.

## Data

We analyze original panel survey data collected in three periods from a sample of adult Jewish Israelis: March–April 2023 (*n* = 1,552), October 2003 (*n* = 1,012), and December 2023 (after the IDF’s ground operation in Gaza began; *n* = 969).^3^ Panel4All, a professional survey company, recruited the respondents from its database of panelists. The initial quota sample was matched to the general population by gender, age, religiosity level, and region (see Appendix A for additional information on sampling, recruitment procedures, and measurement of key variables). Because reinterview rates were not homogeneous (e.g., higher for women than for men), the panel data were analyzed using inverse probability weights, which rematched the panel data to the general population. Because attitudes were measured on rating scales, we used ordered logistic regressions to model attitudinal outcomes. Data analysis was conducted with Stata.

## Findings

The findings are discussed in two parts. The first part examines the distributions of key outcome variables across data collection periods to determine which of two contrasting scenarios—“rally” or “polarization”—emerged among Jewish Israelis following the October 7 attack. The second part tests the “split attitudes” model (figure 1) by examining the associations between outcome variables and predictors, the relationships involving moderators, and the statistical significance of the hypothesized mediation pathways.

### Adjudicating Between the “Rally” and “Polarization” Hypotheses

**Figure 2.**
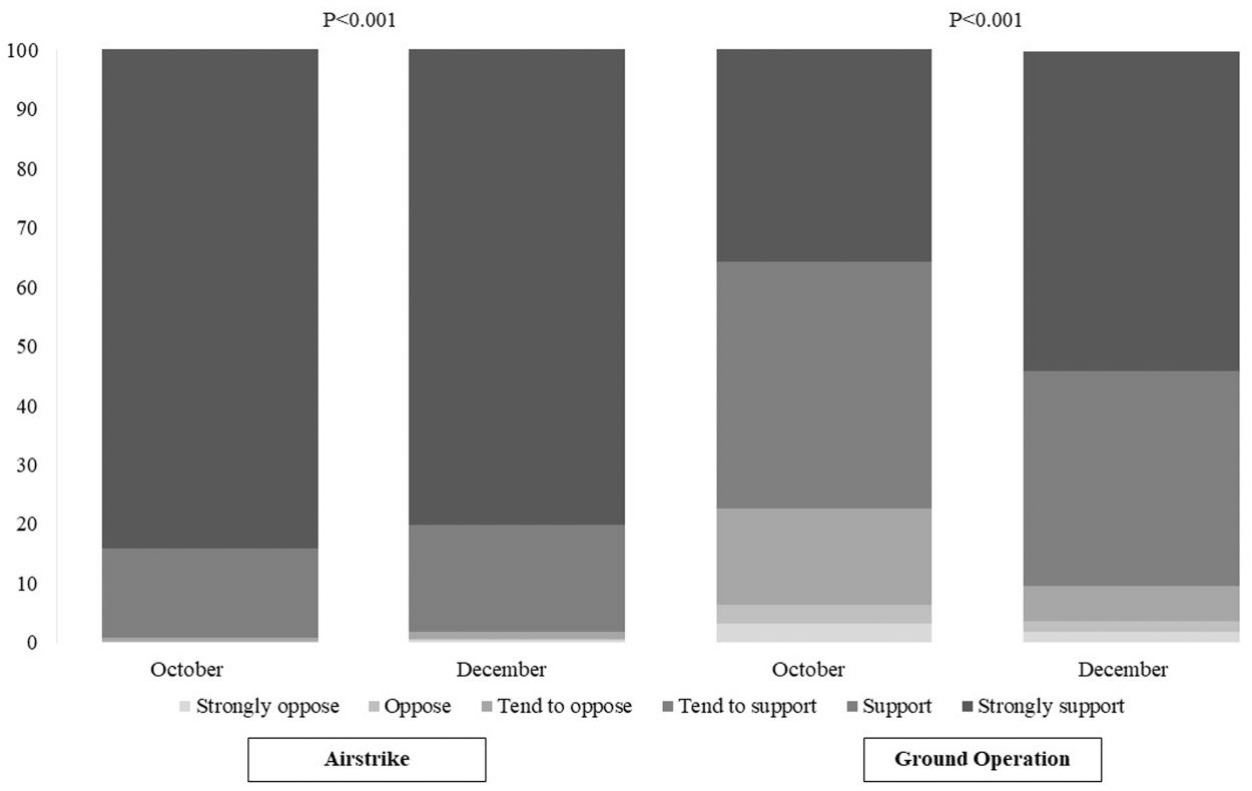
Distribution of support and opposition to the military assault on Gaza in October and December 2023 (*N* = 933).

**Figure 3.**
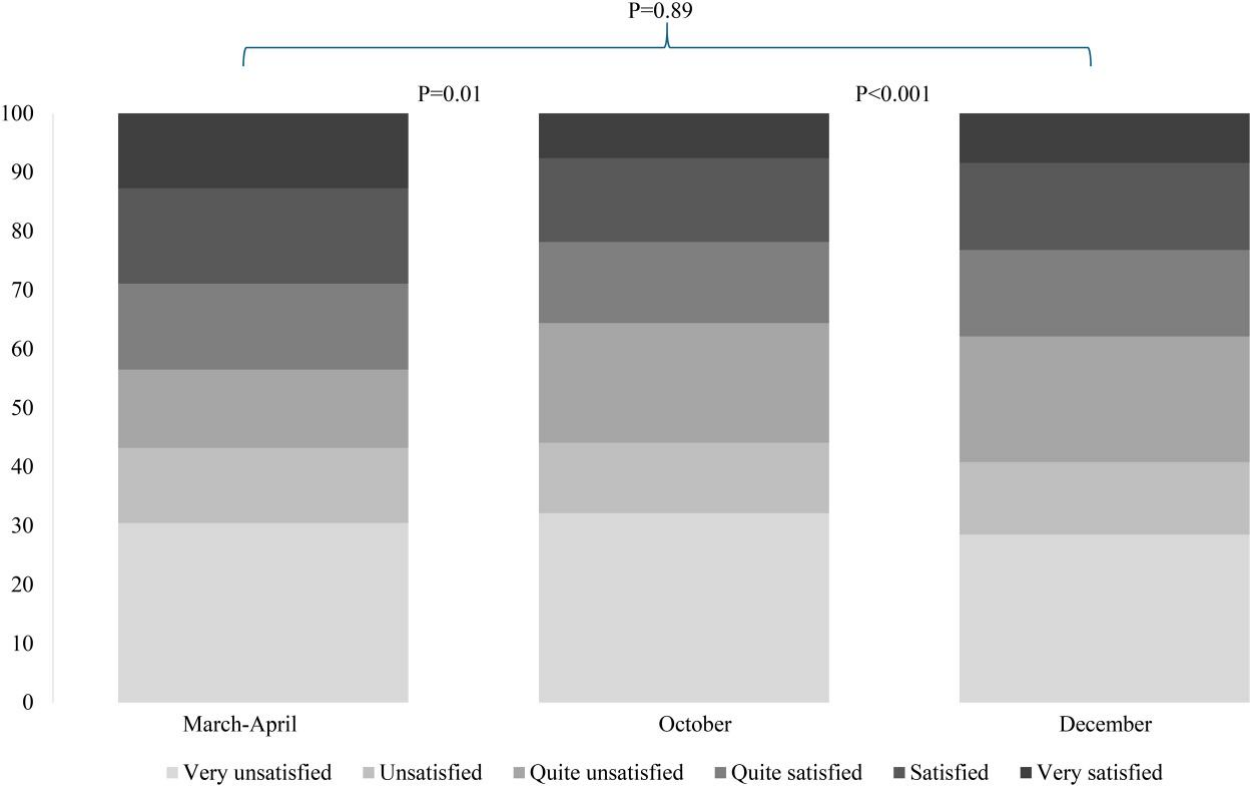
Distribution of satisfaction and dissatisfaction with Prime Minister Netanyahu in March–April, October, and December 2023 (*N* = 939).

**Figure 4.**
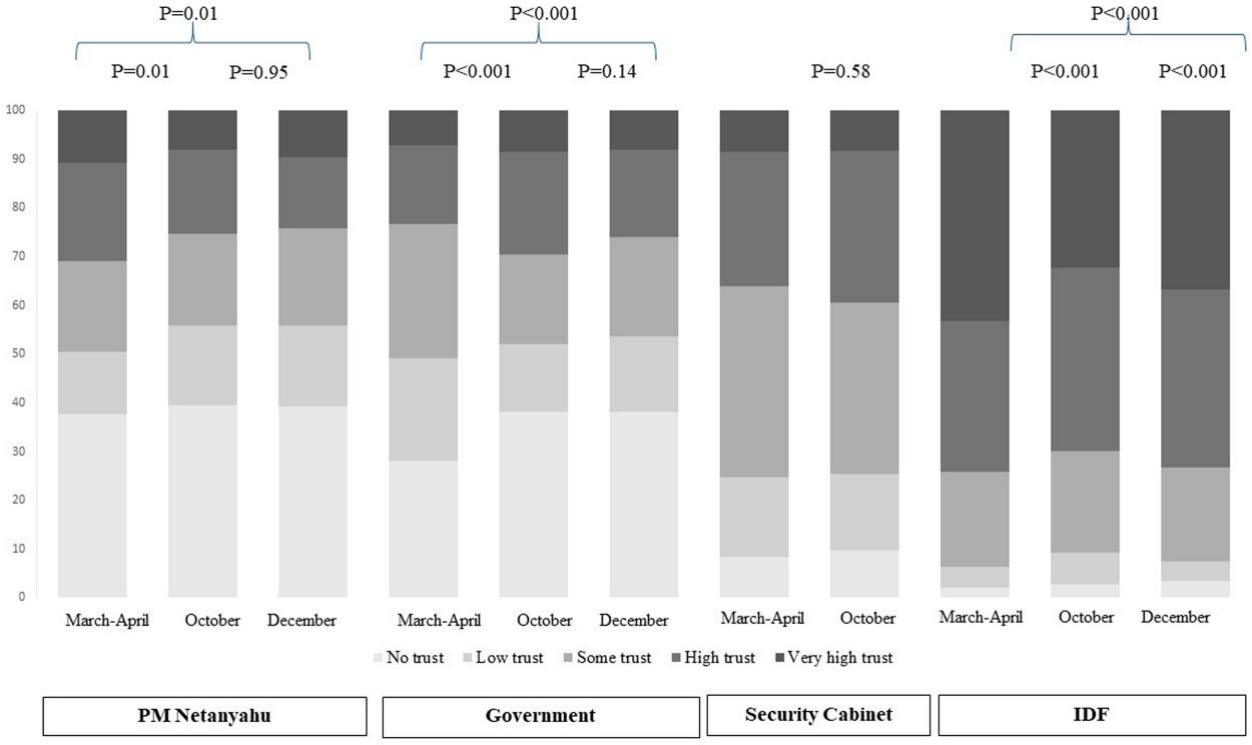
Distributions of trust and distrust in the prime minister, government, security cabinet, and IDF in March–April, October, and December 2023 (*N* = 901).

Figures 2–4 examine the distributions of outcome variables and assess the statistical significance of their changes over time using the Wilcoxon matched-pairs signed-rank test. The columns in the figures compare the weighted distributions of focal variables within the panel sample of participants in all three survey waves.

Figure 2 shows very high levels of support for the military operation in Gaza. Nearly all Jewish Israelis supported the airstrike in Gaza, and a solid majority also supported the ground assault. Initially, support for sending soldiers to fight in Gaza was modest, but it increased significantly when the ground operation began.

Figure 3 shows that, in contrast to the high levels of support for the military operation in Gaza ordered by Netanyahu’s government, satisfaction with the prime minister himself remained low following the October 7 attack. We estimate that only about one-third of the Jewish population in Israel was at least somewhat satisfied with Netanyahu during that period, and less than 10 percent were very satisfied. Satisfaction rates increased slightly when the ground operation in Gaza began, but overall, they did not differ significantly from those measured during the highly divisive period of March–April 2023. In contrast to public reactions during the 2014 Gaza war (Feinstein 2018), there was no rallying behind the prime minister’s leadership during the 2023 war, as satisfaction rates did not exceed those measured before the war.

The split patterns also appear in figure 4, which shows considerable differences between public trust in PM Netanyahu and the government and trust in security institutions. The IDF and the Security Cabinet—which, following the October 7 attack, was expanded to include two former IDF chiefs of staff, Benny Gantz and Gadi Eisenkot, leaders of the HaMahane HaMamlakhti party (roughly translated as *The National Unity Camp*)—enjoyed relatively high public trust. When comparing the data on trust in the IDF across the three survey periods, we observe a slight decline in October, likely due to the shock caused by the surprise attack, and a slight increase in December. However, trust in the IDF was the highest of all the institutions covered by the questionnaire. In contrast, trust in the prime minister and the government was relatively low across the survey periods and, contrary to the “rally” hypothesis, did not increase in response to the security crisis and war.

### Testing the Split Attitudes Model

We begin evaluating the “split attitudes” model by testing the direct associations between the primary predictors and the attitudinal outcomes. Because previous studies have demonstrated that terrorism elicits both anger and fear—and that fear, in contrast to anger, tends to increase risk aversion and reduce support for military action (Lerner and Keltner 2001; Lerner et al. 2003; Skitka et al. 2006; Huddy, Feldman, and Cassese 2007)—we examine the associations of both emotions with the outcome variables. In this section as well, we analyze the panel data consisting of participants from all three survey waves.

Table 1 presents results from ordered logistic regressions of data from the October and December 2023 surveys. Each pair of columns examines the associations between focal predictors and a specific outcome variable. Models 1–6 examine attitudes toward the war and the military, while Models 7–12 examine attitudes toward the prime minister and the government.

**Table 1.** Regression models predicting support for military action; trust in the IDF, government, and prime minister; and satisfaction with the prime minister in October and December 2023 (standard errors in parentheses; *p*-values below, two-tailed tests).

	1	2	3	4	5	6	7	8	9	10	11	12
	Support for an airstrike		Support for ground assault		Trust in the IDF		Satisfied with the PM		Trust in the PM		Trust in the government	
	Oct	Dec	Oct	Dec	Oct	Dec	Oct	Dec	Oct	Dec	Oct	Dec
Anger toward Hamas	3.937	3.637	0.481	1.211	1.589	1.919	−1.491	−1.673	−0.145	−0.899	−1.429	−0.804
	(0.863)	(0.688)	(0.607)	(0.549)	(0.596)	(0.641)	(0.534)	(0.621)	(0.775)	(0.801)	(0.739)	(0.767)
	0.000	0.000	0.428	0.028	0.008	0.003	0.005	0.007	0.851	0.262	0.053	0.295
Fear of Hamas	−1.614	−0.527	−1.065	−0.297	−0.297	−0.421	−0.076	−0.113	−0.181	−0.209	0.360	−0.013
	(0.434)	(0.484)	(0.294)	(0.310)	(0.310)	(0.289)	(0.314)	(0.325)	(0.321)	(0.333)	(0.312)	(0.306)
	0.000	0.276	0.000	0.338	0.338	0.146	0.808	0.729	0.573	0.531	0.248	0.965
Government responsibility	0.196	0.040	−0.006	−0.002	−0.039	0.006	−0.420	−0.506	−0.336	−0.421	−0.367	−0.402
	(0.096)	(0.091)	(0.067)	(0.078)	(0.068)	(0.072)	(0.068)	(0.069)	(0.080)	(0.076)	(0.068)	(0.077)
	0.041	0.659	0.932	0.978	0.563	0.934	0.000	0.000	0.000	0.000	0.000	0.000
Protest responsibility	0.113	0.172	0.032	0.014	−0.070	−0.053	0.099	0.288	0.086	0.307	0.092	0.353
	(0.085)	(0.073)	(0.063)	(0.060)	(0.058)	(0.070)	(0.058)	(0.062)	(0.066)	(0.070)	(0.070)	(0.067)
	0.187	0.020	0.611	0.811	0.228	0.446	0.090	0.000	0.189	0.000	0.188	0.000
Hamas is a threat to Israel	0.632	0.223	0.327	0.244	0.140	0.201	0.194	0.119	0.226	−0.169	0.080	0.175
	(0.162)	(0.143)	(0.119)	(0.123)	(0.093)	(0.142)	(0.137)	(0.149)	(0.125)	(0.128)	(0.116)	(0.147)
	0.000	0.120	0.006	0.048	0.133	0.159	0.157	0.427	0.071	0.186	0.491	0.235
Support of the judicial reform (in March–April)	0.106	0.168	0.024	−0.007	−0.090	−0.076	0.436	0.487	0.515	0.474	0.632	0.549
	(0.086)	(0.066)	(0.051)	(0.056)	(0.048)	(0.053)	(0.065)	(0.059)	(0.065)	(0.060)	(0.068)	(0.063)
	0.219	0.011	0.644	0.906	0.060	0.152	0.000	0.000	0.000	0.000	0.000	0.000
Controls	Yes	Yes	Yes	Yes	Yes	Yes	Yes	Yes	Yes	Yes	Yes	Yes
*F*-test	31.69	37.75	3.91	3.22	1.80	3.67	16.02	17.71	13.08	16.39	18.61	15.08
	0.000	0.000	0.000	0.000	0.009	0.000	0.000	0.000	0.000	0.000	0.000	0.000
*N*	938	932	938	932	927	923	938	932	925	920	921	920

*Note*: Casewise deletion was used in all models. For the coefficients of age, gender, religiosity, education, political orientation, and region, see Appendix B  table B3.

The findings in table 1 reveal markedly different patterns for the two halves of the table, consistent with the split attitudes model. Anger is positively associated with support of the airstrike and trust in the IDF, and also with support for a ground assault in Gaza (the latter association is statistically significant only in December, after the ground operation began). In line with previous studies, fear is associated with *less* militant attitudes, but this effect is statistically significant only in the October data.

Anger has negative associations with all dependent variables in Models 7–12 (but they are statistically significant only in half of the models). The most consistent predictors of satisfaction or dissatisfaction with Netanyahu and trust or distrust in the PM and government are whether respondents attribute responsibility for the October 7 attack to the government, their views on the government-led judicial reform, and—especially in the December survey—whether they attribute responsibility for the crisis to the anti-reform protest.

The split attitudes model distinguishes not only between two sets of outcomes—support for military action and trust in the military on the one hand, and trust in the government as well as trust and satisfaction with the prime minister on the other—but also posits two distinct pathways to these outcomes, each mediated by distinct variables. The path to the first set of outcomes runs through anger, while the path to the second set involves attributing responsibility for Hamas’s attack either to the government or to the protesters. Therefore, table 2 examines the associations between the primary independent variables—perceived threat by Hamas and views on judicial reform—and variables suspected to mediate their effects on the focal outcome variables.

**Table 2.** Regression models predicting anger, fear, and attributing responsibility for the security crisis to the government or protesters, October 2023 (standard errors in parentheses; *p*-values below, two-tailed tests).

	1	2	3	4
	Anger toward Hamas	Fear of Hamas	Responsibility of government	Responsibility of protest
Hamas is a threat to Israel	0.038	0.121	0.065	0.089
	(0.012)	(0.015)	(0.130)	(0.128)
	0.001	0.000	0.616	0.487
Support for the judicial reform (in March–April)	−0.003	−0.001	−0.243	0.372
	(0.003)	(0.007)	(0.049)	(0.053)
	0.269	0.883	0.000	0.000
Controls	Yes	Yes	Yes	Yes
*F*-test	2.68	1.08	6.85	9.35
	0.006	0.000	0.000	0.000
*N*	938	938	939	939

*Note*: OLS regression in Models 1–2 and ordered logistic regression in Models 3–4. Casewise deletion was used in all models. For the coefficients of age, gender, religiosity, education, political orientation, and region, see Appendix B  table B4.

The findings in table 2 support our hypotheses about two separate paths to attitude formation. Anger and fear are positively associated with security threat concerns, which have no significant association with views about who was responsible for the October 7 attack. In contrast, the responsibility variables are associated with views on judicial reforms (supporters of the reform held the protesters responsible, while opponents held the government responsible), but these variables have no statistically significant associations with respondents’ assessments of the threat Hamas posed.

In the final analysis step, we estimated structural equations with direct and indirect paths from the independent variables to the dependent variables, and we used Hayes’s (2009) bootstrapping method with 5,000 repetitions to test the statistical significance of the indirect paths. The results (see Appendix B  table B1) confirmed the statistical significance of the hypothesized mediation paths (except for support for the ground operation that became significant after the operation began). The associations between security threat assessments and attitudes toward the war and the military were mediated by anger, whereas attributions of responsibility for the crisis mediated the relationship between respondents’ views of the judicial reform in March–April 2023 and their evaluations of the government during the war.

## Discussion

The Israel-Hamas war—the most vicious and deadly phase in the protracted Israeli-Palestinian conflict—began on October 7, 2023, with the brutal attack by Hamas on civilian localities and military installations along the Israel-Gaza border, followed by the most massive, prolonged, deadly, and destructive operation by the IDF in Gaza. In response to the crisis, a majority of Jewish Israelis supported the decision to declare war, and their confidence in the military was only slightly and temporarily eroded by the failure to prevent the October 7 attack. In contrast, public assessments of the government and prime minister remained as polarized as before the security crisis and war.^4^

Our analysis of the correlates of two sets of attitudes—toward the war and security forces, and toward the government and prime minister—revealed two distinct patterns. Similar to previous research on public reactions to security crises and wars, support for military action and trust in the national security forces were associated with anger about the actions of enemies held responsible for the nation’s security crisis. In contrast, people’s views of the government were not associated with anger toward the enemy but with their views on the government’s judicial reform initiative and the protest movement against it: those who supported the reform expressed relatively high trust in the government and prime minister and blamed the protesters for the security crisis, whereas opponents of the reform distrusted the prime minister and government, whom they held responsible for the crisis.

Therefore, our primary conclusion, which requires further examination in different countries and contexts, is that extreme levels of polarization limit the potential for a sweeping rally-’round-the-flag response to a security crisis. We highlight a particular aspect of polarization as responsible for the complete absence of a rally behind the prime minister and the government. During the 2014 Gaza war, high levels of affective polarization—steadily rising since Netanyahu’s return to office in 2009 (Amitai, Gidron, and Yair 2023)—likely weakened the rally effect, with Netanyahu’s favorability among Jewish Israelis reaching only about two-thirds (Feinstein 2018). This pattern was largely driven by the reluctance of left- and center-left identifiers to support his leadership even during wartime. However, in our October 2023 survey, 67 percent of respondents identified with the political right. Based on this distribution alone, we would have expected at least a modest rally behind Netanyahu’s leadership during one of the most intense wars in Israel’s history. We suggest that the complete absence of a rally was caused by the polarizing effect of the government’s judicial reform initiative, which eroded trust not only among the left and center but also among parts of the right.

We further propose that the absence of a significant rally behind the government in response to the security crisis and war can be largely explained by blame attribution. When citizens hold the government responsible for a crisis, a rally effect is unlikely. Evidence for this dynamic was also found in studies of public reactions in Spain to the March 11, 2004, Madrid bombings, where many voters blamed the government’s support for the 2003 Iraq invasion, which contributed to the electoral upset (Bali 2007; Tena-Sánchez 2018).

Although generalization cannot be made based on a single case, our findings suggest—and we propose as a hypothesis for future research—that attacks by populist authoritarian leaders on democratic institutions may not only increase political polarization and deepen citizens’ distrust and disdain toward rival parties and leaders (Gessler and Wunsch 2025) but also, as a result, significantly limit governments’ ability to mobilize rallies behind their leadership even in times of war. Israel is not unique in facing internal struggles over the nature of its regime. However, among the countries currently experiencing such conflicts, Israel likely faces the most severe security threats and is engaged in relatively frequent and intense military confrontations. We therefore view Israel and the October 7 crisis and subsequent war as an extreme case, suggesting that in other countries experiencing similar internal strife, the likelihood of a massive rally behind the government is even lower than in Israel.

Finally, while our study focused on the initial stages of the Hamas-Israel war, it is important to note that low trust in the government and prime minister persisted throughout the conflict (aChord 2024). Even before the fighting ended, mass protests reemerged, driven by divisions over whether the government’s handling of ceasefire negotiations—potentially leading to the release of hostages—was influenced by political considerations. Supporters of the government rejected this view, arguing that Hamas’s hardline stance prevented a reasonable agreement and that a poor deal would have left Hamas entrenched in Gaza. This ongoing polarization illustrates a broader dynamic: whereas previous research has shown that crises often produce a temporary rally effect that later gives way to renewed polarization (e.g., Jacobson 2007), our findings suggest that in highly polarized societies, even major security crises may reinforce existing divisions from the outset. When preexisting political cleavages remain salient, and debates over responsibility and government performance quickly take center stage, the struggle over the interpretation of events (Reed 2006) perpetuates political divides. However, caution is needed when setting expectations about the longer-term effects of security crises and wars: whether polarization ultimately increases or declines (or remains stable) may depend heavily on subsequent struggles over the collective memory and legacy of events—an issue we leave open for future research.

## Supplementary Material

nfag014_Supplementary_Data

## Data Availability

Replication data and documentation are available at https://dataverse.harvard.edu/dataset.xhtml?persistentId=doi%3A10.7910%2FDVN%2FUYYEXD&version=DRAFT.

## Footnotes

1 Emotions influenced government assessments during data collection, even though absent from the model’s upper branch (figure 1). The model suggests that anger drives attitudes during rallies, but has a lesser impact when controversial policies shape views. A more advanced model and detailed data could better capture emotions triggered by such policies.

2 By “substantial increase” we mean a change of more than a few percentage points—since smaller changes may result from various factors that do not reflect a meaningful shift in public mood or could even be due to sampling errors (Feinstein 2016, 352).

3 Arab/Palestinian citizens of Israel were not included in the sample for technical and substantive reasons. Technically, they were not part of the larger panel survey providing our baseline data, and extending the sample within the crisis onset timeframe was infeasible. Substantively, after October 7, many faced threats of scapegoating and hostility, likely leading to high refusal rates and self-censorship that would compromise data quality. Additionally, our theoretical framework is less applicable to this “trapped minority” (Rabinowitz 2001), who historically have not rallied behind wars between Israel and Palestinian factions—a pattern that polls suggest continued in October 2023.

4 A potential limitation of this study is its use of a nonprobability sample, which could bias the results if participation is related to respondents’ attitudes on the focal variables; Appendix A presents evidence mitigating this concern.

## Appendix A. AAPOR-Required Disclosure Elements

Panel4All maintains a dynamic database of over 30,000 active participants per year and adheres to the highest standards in online polling, particularly those designed to prevent various forms of recruitment and response bias (see www.panel4all.com/images/panel/panel4all_esomar26.pdf). Each month, approximately 1,000 new participants are recruited through online campaigns (e.g., social media and web-based advertising), referral programs, and targeted outreach efforts aimed at hard-to-reach social groups. The multistage recruitment process is designed to validate participant identity and ensure voluntary, double opt-in enrollment.

For each wave of data collection in this study, potential participants were invited via email to complete the survey using their mobile phones or personal computers. The cooperation rate in the March–April 2023 survey was 28 percent. The reinterview rates were 71 percent in the October 2023 survey and 93 percent in the December 2023 survey. Each questionnaire included one or two filtering questions to identify and remove “speeders” who answered without reading the questions—across the three survey waves, 6–8 percent of participants failed these filters and were not included in the dataset. Overall, the attrition rates were 35 percent between April and October 2023 and 4 percent between October and December 2023.

Most questions focused on beliefs and attitudes and were answered on rating scales. The March–April survey (conducted March 26–April 4 and April 25–26) included 95 questions and was completed by 1,552 participants, with a median completion time of 14 minutes; the October survey (conducted October 18–22) included 65 questions and was completed by 1,010 participants, with a median time of 18 minutes; and the December survey (conducted December 4–14) comprised 45 questions and was completed by 966 participants, with a median time of 10 minutes. Upon completion, participants received minimal tokenistic compensation—ranging from approximately one-quarter to one US dollar for a 10–20-minute survey—delivered as a gift card.

A potential limitation of this study is its reliance on a nonprobability sample, chosen for its speed of data collection and high reinterview rates. Such sampling carries a risk of bias if respondents’ likelihood of participation is associated with their attitudes. Although this risk cannot be entirely ruled out, three considerations support the validity of our primary findings regarding split attitudes. First, although motivation to participate in the baseline survey (March–April 2023) may have been influenced by views on the judicial reform initiative and the government, attitudes in our sample were nearly evenly divided. A few surveys conducted during the same period reported greater opposition to the reform (Hermann and Anabi 2023; Tachlith Institute 2023). Therefore, to the extent that any bias is present, it likely overrepresents supporters of the reform and the government, making our estimates of high disapproval rates of the government following the October 7 attack conservative. Second, repeated participation in our surveys did not vary systematically with views about judicial reform or partisanship. It is not possible to determine whether the likelihood of repeated participation was associated with attitudes toward the security crisis and the war; however, this potential limitation should be comparable in a panel survey drawn from an initial probabilistic sample. Third, the observed split attitudes pattern at the center of our discussion is unlikely to be an artifact of the sampling or recruitment procedures, as similar methods in earlier research yielded markedly different patterns during a rally-’round-the-flag period in Israel (Feinstein 2018).

**Key Variables Measurement** 

***Dependent variables***

Support for airstrikes and ground assaults was measured using the questions: “To what extent do you support or oppose an airstrike/ground assault in Gaza?” Responses were recorded on a rating scale with six categories, ranging from *strongly oppose* to s*trongly support*.

Trust in institutions and personnel—including the government, the prime minister, the IDF, and the security cabinet—was measured using the question: “Generally speaking, how much trust do you have in the following organizations and individuals?” Responses were recorded on a rating scale with five categories, ranging from *no trust at all* to *very high trust*. The list of options included “I do not know,” which we treated as missing values in the analysis. We note that only 1–2 percent of respondents selected this option, except for the security cabinet, with 4 percent missing.

Satisfaction with the prime minister was assessed by asking: “To what extent are you satisfied with Prime Minister Netanyahu’s performance?” Responses were recorded on a rating scale with six categories, ranging from *very unsatisfied* to *very satisfied*.

***Independent variables***

Perceptions of threat to Israel by Hamas were measured using the question: “To what extent do you agree or disagree with the following statement: Hamas poses a security threat to the state of Israel?” Responses were recorded on a rating scale with five categories, ranging from *strongly not at all agree* to *very much agree*.

Views of the judicial reform were measured by asking: “To what extent do you support or oppose the judicial reform?” Responses were recorded on a rating scale with seven categories, ranging from *strongly oppose* to *strongly support*. The list of answers included two additional options: “I am not sure what my opinion is” and “I am not familiar with the issue” (6 and 1 percent selected these options, respectively). In the regression analyses, we treat these responses as missing values. However, recoding the “not sure” answer into the middle category (“neither agree nor disagree”) does not significantly alter the findings.

***Mediators***

Attributing responsibility for the security crisis to the government or the protests against the judicial reform was measured by asking: “To what extent, if at all, are the following bodies responsible for Hamas’s decision to attack the Gaza border communities?” One item referred to the government, and the other to the protesters. Responses were recorded on a rating scale with five categories, ranging from *not at all* to *to a very large extent*.

**Feelings About Hamas** 

We estimated levels of anger and fear as composite measures, each constructed from a set of survey items sharing a latent factor. The scores were standardized, averaged, and then rescaled to range from 0 to 1. The following prompt assessed feelings about Hamas: “Below are several statements referring to your feelings when you think about Hamas in Gaza. Please indicate the extent to which you agree or disagree with each statement.” Respondents were asked to indicate their level of agreement with emotionally framed statements, using a seven-point scale ranging from *not at all agree* to *strongly agree*, with statements containing emotional labels: “Hamas makes me feel [emotion label].” The anger scale was composed of “anger,” “loathing,” and “disgust” (Cronbach’s alpha = 0.86), while the fear scale included items on “fear” and “anxiety” (Cronbach’s alpha = 0.93).

## Appendix B. Additional Analysis Tables

**Table B1.** Testing indirect (mediated) effects.

	Mediation path	95% Confidence interval	
October	Threat->Anger->Support air strike	0.069	0.302
	Threat->Anger->Support ground assault	−0.024	0.079
	Threat->Anger ->Trust in the IDF	0.009	0.125
	Reform->Responsibility->Satisfaction by the prime minister	0.128	0.420
	Reform->Responsibility->Trust in the prime minister	0.040	0.308
	Reform->Responsibility->Trust in the government	0.096	0.389
December	Threat->Anger->Support airstrike	0.077	0.370
	Threat->Anger->Support ground assault	0.010	0.138
	Threat->Anger->Trust in the IDF	0.030	0.182
	Reform->Responsibility->Satisfaction by the prime minister	0.138	0.440
	Reform->Responsibility->Trust in the prime minister	0.064	0.357
	Reform->Responsibility->Trust in the government	0.085	0.355

**Table B2.** Variable distributions in the three 2023 survey waves.

Variable	Categories	Mar–Apr	Oct	Dec	Variable	Categories	Mar–Apr	Oct	Dec
Support airstrike	Strongly oppose		0.14	0.44	Trust in government	No trust	27.98	38.07	38.14
	Oppose		0.05	0.26		Low trust	21.25	13.89	15.43
	Tend to oppose		0.6	1.21		Some trust	27.46	18.42	20.41
	Tend to support		3.73	4.48		High trust	16.15	21.16	18.09
	Support		11.38	13.41		Very high trust	7.16	8.47	7.93
	Strongly support		84.1	80.2					
Support ground assault	Strongly oppose		3.13	2.07	Support/oppose the judicial reform	Strongly oppose	24.72	24.39	
	Oppose		3.35	1.82		Oppose	9.85	10.72	
	Tend to oppose		16.16	6.02		Tend to oppose	4.95	5.66	
	Tend to support		22.12	13.92		Neither support nor oppose	13.7	8.14	
	Support		19.51	22.32		Tend to Support	9.39	7.62	
	Strongly support		35.73	53.86		Support	15.08	11.68	
						Strongly support	22.3	24.47	
Trust in the IDF	No trust	2	2.72	3.44	Assessment of Hamas’s threat to Israel’s security	Strongly disagree		0.72	0.55
	Low trust	4.38	6.44	4.02		Disagree		1.7	0.71
	Some trust	19.41	20.9	19.32		Neither agree nor disagree		3.38	5.23
	High trust	31	37.66	36.42		Agree		13.7	15.32
	Very high trust	43.2	32.27	36.8		Strongly disagree		80.49	78.18
Prime minister satisfaction	Very unsatisfied	30.47	32.14	28.5	How much is the government responsible for the attack?	Not responsible at all		10.11	11.38
	Unsatisfied	12.75	11.98	12.29		Not so much		9.03	12.61
	Quite unsatisfied	13.3	20.3	21.35		Responsible to some extent		16.02	18.15
	Quite satisfied	14.61	13.72	14.64		Responsible		26.12	29.86
	Satisfied	16.16	14.26	14.77		Highly responsible		38.73	27.99
	Very satisfied	12.71	7.6	8.45					
Trust in prime minister	No trust	37.62	39.48	39.37	How much is the protest responsible for the attack?	Not responsible at all		24.63	22.91
	Low trust	12.99	16.45	16.57		Not so much		15.17	15.43
	Some trust	18.48	18.85	19.79		Responsible to some extent		17.22	19.57
	High trust	20.1	17.23	14.57		Responsible		15.77	17.29
	Very high trust	10.81	7.99	9.69		Highly responsible		27.21	24.81

**Table B3.** Unstandardized coefficients of demographic variables omitted from table 1 (standard errors in parentheses; *p*-values below, two-tailed tests).

	1	2	3	4	5	6	7	8	9	10	11	12
	Support for an airstrike		Support for ground assault		Trust in IDF		Satisfied with the PM		Trust in the PM		Trust in the government	
	Oct	Dec	Oct	Dec	Oct	Dec	Oct	Dec	Oct	Dec	Oct	Dec
Age	0.019	0.018	0.008	0.017	0.020	0.031	0.009	0.016	0.013	0.018	0.005	0.001
	(0.009)	(0.008)	(0.005)	(0.006)	(0.006)	(0.006)	(0.006)	(0.006)	(0.006)	(0.006)	(0.007)	(0.006)
	0.033	0.037	0.164	0.004	0.001	0.000	0.095	0.003	0.033	0.003	0.402	0.897
Male	0.364	0.264	0.864	0.479	−0.383	−0.356	0.030	−0.100	−0.005	−0.133	−0.004	−0.149
	(0.252)	(0.241)	(0.168)	(0.179)	(0.165)	(0.162)	(0.166)	(0.163)	(0.171)	(0.173)	(0.177)	(0.174)
	0.149	0.275	0.000	0.008	0.020	0.028	0.856	0.538	0.978	0.440	0.981	0.390
Religiosity: Secular												
Observant	0.348	0.093	0.142	−0.290	0.116	0.144	0.066	−0.127	−0.014	−0.106	−0.217	0.121
	(0.343)	(0.286)	(0.187)	(0.206)	(0.183)	(0.188)	(0.184)	(0.195)	(0.193)	(0.212)	(0.220)	(0.214)
	0.311	0.744	0.446	0.159	0.526	0.444	0.721	0.515	0.942	0.618	0.322	0.574
Religious	0.090	−0.370	0.032	−0.072	−0.083	−0.502	−0.041	−0.152	0.056	−0.189	0.139	0.214
	(0.467)	(0.409)	(0.261)	(0.298)	(0.256)	(0.254)	(0.268)	(0.257)	(0.259)	(0.271)	(0.262)	(0.269)
	0.848	0.366	0.903	0.810	0.745	0.049	0.877	0.555	0.830	0.485	0.597	0.426
Orthodox	−0.225	−0.341	0.539	0.197	−0.049	−0.477	0.925	1.072	0.573	0.967	0.458	0.369
	(0.535)	(0.465)	(0.355)	(0.522)	(0.301)	(0.393)	(0.377)	(0.323)	(0.379)	(0.341)	(0.358)	(0.313)
	0.674	0.464	0.129	0.706	0.872	0.225	0.014	0.001	0.131	0.005	0.201	0.238
Education: Elementary school												
High school, including incomplete	−13.052	−12.899	0.909	−0.610	−1.341	−1.587	0.656	−1.612	0.453	−1.379	−0.289	−1.634
	(0.686)	(0.633)	(0.439)	(1.267)	(1.251)	(1.357)	(0.661)	(1.757)	(0.802)	(1.859)	(0.758)	(1.346)
	0.000	0.000	0.039	0.631	0.284	0.243	0.321	0.359	0.572	0.458	0.703	0.225
Secondary	−13.476	−12.293	1.134	−0.293	−1.356	−2.146	0.447	−2.038	0.103	−1.969	−0.142	−1.827
	(0.722)	(0.609)	(0.446)	(1.244)	(1.260)	(1.351)	(0.655)	(1.752)	(0.817)	(1.861)	(0.792)	(1.356)
	0.000	0.000	0.011	0.814	0.282	0.112	0.495	0.245	0.900	0.290	0.858	0.178
Academic, BA	−12.376	−12.553	1.280	−0.235	−1.402	−2.178	0.163	−1.827	0.025	−1.754	−0.572	−1.855
	(0.696)	(0.613)	(0.447)	(1.257)	(1.262)	(1.359)	(0.655)	(1.748)	(0.812)	(1.858)	(0.752)	(1.346)
	0.000	0.000	0.004	0.852	0.267	0.109	0.804	0.296	0.976	0.345	0.447	0.169
Academic, graduate degree	−13.012	−12.434	1.371	−0.181	−1.631	−2.205	−0.118	−2.435	−0.224	−2.160	−0.855	−2.263
	(0.725)	(0.621)		(1.261)	(1.259)	(1.355)	(0.666)	(1.757)	(0.815)		(0.766)	(1.359)
	0.000	0.000	0.003	0.886	0.195	0.104	0.859	0.166	0.783	0.247	0.265	0.096
Political orientation: Left												
Moderate left	1.394	1.291	0.544	0.821	0.423	0.941	−0.291	−0.396	−1.107	0.093	−0.023	0.566
	(0.513)	(0.369)	(0.430)	(0.412)	(0.405)	(0.387)	(0.812)	(0.748)	(0.915)	(0.870)	(0.733)	(1.090)
	0.007	0.000	0.206	0.047	0.296	0.015	0.720	0.597	0.227	0.915	0.975	0.603
Center	0.871	1.599	0.509	1.125	0.816	1.204	0.888	0.836	0.482	0.433	0.785	0.792
	(0.458)	(0.384)	(0.409)	(0.338)	(0.405)	(0.372)	(0.762)	(0.738)	(0.818)	(0.865)	(0.627)	(1.058)
	0.058	0.000	0.214	0.001	0.044	0.001	0.244	0.257	0.556	0.617	0.211	0.454
Moderate right	1.726	2.481	0.408	1.527	1.004	1.466	1.406	1.365	0.986	1.262	1.265	1.621
	(0.535)	(0.423)	(0.427)	(0.378)	(0.415)	(0.388)	(0.781)	(0.759)	(0.837)	(0.876)	(0.625)	(1.065)
	0.001	0.000	0.339	0.000	0.016	0.000	0.072	0.073	0.239	0.150	0.043	0.128
Right	2.530	2.696	0.439	1.696	1.383	1.739	1.747	1.633	1.178	1.467	1.405	1.918
	(0.605)	(0.512)	(0.458)	(0.426)	(0.436)	(0.408)	(0.805)	(0.781)	(0.864)	(0.902)	(0.639)	(1.086)
	0.000	0.000	0.338	0.000	0.002	0.000	0.030	0.037	0.173	0.104	0.028	0.078
Region: South												
Haifa	−0.584	−0.009	−0.066	0.153	0.027	−0.038	−0.082	−0.301	0.227	−0.185	−0.299	−0.374
	(0.444)	(0.383)	(0.260)	(0.276)	(0.260)	(0.253)	(0.257)	(0.268)	(0.273)	(0.307)	(0.315)	(0.291)
	0.188	0.982	0.801	0.580	0.916	0.882	0.749	0.262	0.406	0.547	0.343	0.199
West Bank	0.588	0.285	−0.089	0.034	−0.316	−0.424	−1.104	−0.891	−1.093	−0.906	−0.362	−0.998
	(1.142)	(0.701)	(0.381)	(0.439)	(0.456)	(0.425)	(0.526)	(0.438)	(0.530)	(0.444)	(0.396)	(0.473)
	0.607	0.684	0.816	0.939	0.489	0.318	0.036	0.042	0.039	0.042	0.360	0.035
Jerusalem	−1.131	−0.089	−0.070	0.089	−0.002	−0.193	−0.224	−0.216	−0.136	−0.027	0.116	0.119
	(0.440)	(0.461)	(0.300)	(0.348)	(0.303)	(0.314)	(0.309)	(0.362)	(0.338)	(0.345)	(0.349)	(0.333)
	0.010	0.847	0.815	0.797	0.995	0.540	0.468	0.551	0.687	0.938	0.739	0.722
Center	−0.364	0.444	0.217	0.070	−0.270	−0.122	−0.551	−0.324	−0.364	−0.132	−0.375	−0.477
	(0.412)	(0.345)	(0.231)	(0.250)	(0.215)	(0.215)	(0.240)	(0.248)	(0.246)	(0.267)	(0.257)	(0.257)
	0.377	0.198	0.349	0.780	0.210	0.571	0.022	0.192	0.139	0.620	0.144	0.063
North	0.098	0.206	−0.228	−0.007	0.173	0.360	−0.213	−0.274	0.040	−0.299	0.223	0.151
	(0.514)	(0.413)	(0.355)	(0.330)	(0.379)	(0.326)	(0.371)	(0.360)	(0.327)	(0.441)	(0.460)	(0.488)
	0.849	0.618	0.521	0.983	0.648	0.269	0.565	0.447	0.902	0.498	0.628	0.756
Tel Aviv	−0.117	0.029	−0.008	−0.084	−0.290	−0.292	−0.085	−0.120	−0.140	−0.116	−0.209	−0.245
	(0.433)	(0.341)	(0.249)	(0.261)	(0.233)	(0.236)	(0.237)	(0.257)	(0.256)	(0.304)	(0.286)	(0.303)
	0.787	0.933	0.975	0.747	0.214	0.217	0.721	0.640	0.585	0.703	0.466	0.418
Constant 1	−1.920	−1.307	0.253	−0.033	−2.510	−1.246	0.521	−1.935	2.557	−1.283	0.577	0.419
	(1.662)	(1.204)	(0.896)	(1.449)	(1.494)	(1.554)	(1.188)	(2.020)	(1.405)	(2.206)	(1.307)	(1.914)
	0.000	0.000	0.778	0.982	0.093	0.423	0.661	0.338	0.069	0.561	0.659	0.827
Constant 2	−1.605	−9.832	1.029	0.622	−1.203	−0.409	1.412	−0.891	3.736	−0.087	1.768	1.738
	(1.583)	(1.155)	(0.895)	(1.459)	(1.484)	(1.532)	(1.186)	(2.025)	(1.426)	(2.201)	(1.305)	(1.926)
	0.000	0.000	0.251	0.670	0.418	0.790	0.234	0.660	0.009	0.968	0.176	0.367
Constant 3	−9.126	−8.775	2.559	1.648	0.297	1.230	2.852	0.681	5.040	1.359	3.184	3.407
	(1.482)	(1.145)	(0.884)	(1.463)	(1.492)	(1.538)	(1.184)	(2.036)	(1.436)	(2.194)	(1.310)	(1.947)
	0.000	0.000	0.004	0.260	0.842	0.424	0.016	0.738	0.000	0.536	0.015	0.080
Constant 4	−7.194	−7.362	3.680	2.757	1.992	2.962	3.888	1.811	6.787	2.909	5.217	5.384
	(1.407)	(1.128)	(0.889)	(1.475)	(1.494)	(1.532)	(1.187)	(2.032)	(1.456)	(2.187)	(1.320)	(1.948)
	0.000	0.000	0.000	0.062	0.183	0.053	0.001	0.373	0.000	0.184	0.000	0.006
Constant 5	−5.522	−5.753	4.551	3.859			5.432	3.538				
	(1.435)	(1.119)	(0.898)	(1.474)			(1.205)	(2.019)				
	0.000	0.000	0.000	0.009			0.000	0.080				
*F*-test	31.69 .000	37.75 .000	3.91 .000	3.22 .000	1.80 (0.009)	3.67 .000	16.02 .000	17.71 .000	13.08 .000	16.39 .000	18.61 .000	15.08 .000
*N*	938	932	938	932	927	923	938	932	925	920	921	920

**Table B4.** Unstandardized coefficients of demographic variables omitted from table 2 (standard errors in parentheses; *p*-values below, two-tailed tests).

	1	2	3	4
	Anger toward Hamas	Fear of Hamas	Responsibility of government	Responsibility of protest
Age	0.001	−0.002	−0.006	0.009
	(0.000)	(0.001)	(0.006)	(0.006)
	0.044	0.015	0.315	0.121
Male	−0.014	−0.200	−0.302	0.141
	(0.011)	(0.022)	(0.158)	(0.150)
	0.184	0.000	0.056	0.347
Religiosity: Secular				
Observant	0.017	−0.009	0.246	0.380
	(0.015)	(0.026)	(0.172)	(0.186)
	0.242	0.726	0.153	0.042
Religious	0.030	−0.023	−0.552	−0.301
	(0.017)	(0.039)	(0.251)	(0.254)
	0.079	0.560	0.028	0.237
Orthodox	0.028	−0.085	−0.385	−0.539
	(0.018)	(0.052)	(0.369)	(0.344)
	0.124	0.101	0.297	0.117
Education: Elementary school				
High school, including incomplete	−0.038	0.265	1.183	−2.286
	(0.027)	(0.217)	(1.199)	(1.306)
	0.157	0.223	0.324	0.080
Secondary	−0.001	0.255	1.600	−2.297
	(0.025)	(0.218)	(1.211)	(1.317)
	0.957	0.241	0.187	0.081
Academic, BA	−0.008	0.265	1.310	−2.420
	(0.026)	(0.218)	(1.200)	(1.320)
	0.761	0.225	0.275	0.067
Academic, graduate degree	−0.024	0.272	1.491	−2.421
	(0.028)	(0.217)	(1.211)	(1.316)
	0.392	0.211	0.218	0.066
Political orientation: Left				
Moderate left	0.016	−0.019	−0.047	−0.065
	(0.035)	(0.043)	(0.380)	(0.360)
	0.645	0.660	0.901	0.857
Center	0.063	−0.014	−0.311	0.405
	(0.026)	(0.040)	(0.363)	(0.348)
	0.014	0.718	0.391	0.245
Moderate right	0.064	−0.034	−0.566	0.521
	(0.028)	(0.042)	(0.356)	(0.363)
	0.026	0.416	0.112	0.151
Right	0.066	−0.043	−0.469	0.608
	(0.030)	(0.047)	(0.381)	(0.405)
	0.026	0.367	0.219	0.134
Region: South				
Haifa	0.009	−0.004	0.181	0.305
	(0.022)	(0.036)	(0.272)	(0.264)
	0.698	0.918	0.505	0.248
West Bank	0.055	0.035	0.510	−0.038
	(0.021)	(0.054)	(0.593)	(0.370)
	0.008	0.511	0.390	0.919
Jerusalem	0.029	0.047	−0.158	−0.131
	(0.020)	(0.043)	(0.325)	(0.327)
	0.143	0.272	0.627	0.689
Center	0.009	−0.034	0.226	0.074
	(0.019)	(0.036)	(0.238)	(0.244)
	0.651	0.338	0.344	0.762
North	0.041	−0.053	0.323	0.245
	(0.018)	(0.046)	(0.269)	(0.326)
	0.023	0.251	0.230	0.451
Tel Aviv	0.013	0.043	0.343	0.169
	(0.020)	(0.036)	(0.245)	(0.260)
	0.505	0.229	0.162	0.515
Constant 1	0.683	0.061	−2.467	−0.798
	(0.071)	(0.238)	(1.458)	(1.507)
	0.000	0.795	0.091	0.596
Constant 2			−1.656	0.047
			(1.454)	(1.509)
			0.255	0.975
*F*-test	1.08	2.68	6.85	9.35
	0.000	0.006	0.000	0.000
*N*	938	938	939	939
