# Supplementary material for: United or Divided? A Polarized Society’s Response to War
Source: Public Opin Q. 2026 Apr 3;90(3):657–96. doi: 10.1093/poq/nfag014 (PMC13287526; doi:10.1093/poq/nfag014)
Supplement: nfag014_Supplementary_Data [file nfag014_supplementary_data.pdf]

United or Divided? A Polarized Society's Response to War

Supplementary Appendix

Yuval Feinstein, Associate Professor, Department of Sociology, Vice Dean of Research, Faculty of Social Sciences, University of Haifa, Haifa, Israel.

Geffen Ben-David, PhD Candidate, Department of Sociology, University of Haifa, Haifa, Israel.

Contact information

Yuval Feinstein, 7013 Rabin Building, 199 Abba Koushi Avenue 199, Mount Carmel, Haifa 3498838, Israel; fyuval@haifa.aci.il

Contents

Supplemental Analyses of Partisanship, Political Identity, and Support for or Opposition to Israel's Judicial Reform and Concerns About Its Democracy…………………………………….. 2

List of Tables and Figures

Supplementary Material Table 1. Spearman Correlations Between Left–Right Self-Placement and Perceived Threat to Israel's Democracy……………………………………………………… 2

Supplementary Material Figure 1. Views of the judicial reform by period and ideological alignment before and during the war…………………………………………………………… 3

Supplementary Material Figure 2. Views on the judicial reform in October 2023, by party voted for in the November 2022 election and party supported in October 2023……………….. 4

Supplementary Material Figure 3. View of PM Netanyahu in October 2023 among supporters and opposers of the judicial reform in the general sample and voters of Likud and HaTzionut HaDatit……………………………………………………………………………….. 6

Supplemental Analyses of Partisanship, Political Identity, and Support for or Opposition to Israel's Judicial Reform and Concerns About Its Democracy

In our March–April 2023 survey, conducted at the height of the protest against the judicial reform initiative, 46 percent supported the reform, 40 percent opposed it, and 5.5 percent expressed no clear stance. These figures remained nearly unchanged in October 2023, with 43 percent in support, 40 percent opposed, and 8 percent neutral. This pretty even split stood in contrast to the political distribution: in both survey periods, about two-thirds of respondents identified with the right, one-fifth with the center, and one-tenth with the left. Self-positioning on the left-right scale has remained relatively stable across the survey periods, with only about 10 percent of participants shifting between the left, center, and right categories. However, the relationship between ideological positioning and concern about the future of Israeli democracy changed significantly over time, as shown in Supplementary Material Table 1. In May 2022, this association was negligible, but by March–April 2023, a moderate negative correlation had emerged and persisted through October 2023.

Supplementary Material Table 1. Spearman Correlations Between Left–Right Self-Placement and Perceived Threat to Israel's Democracy (Both Measured on 5-Point Scales).

	May 2022	March-April 2023	October 2023
Spearman's r	.026	-.455	-.422
p-value	.431	.0000	.0000

The growing association between concerns about democracy and the left-right divide was driven by increasing worries among the Left and Center about the fate of liberal policies and institutions if the judicial reform succeeds, alongside decreasing concerns among the Right, whose majoritarian view of democracy aligns with the judicial reform initiative (Feinstein 2025). However, views on the judicial reform and its perceived impact on democracy not only aligned with the left–right divide but also created a rift among Jewish Israelis identifying with the

political right, leading to a partisan realignment, as revealed in Supplementary Material Figures 1–3.

Supplementary Material Figure 1. Views of the judicial reform by period and ideological alignment before and during the war.

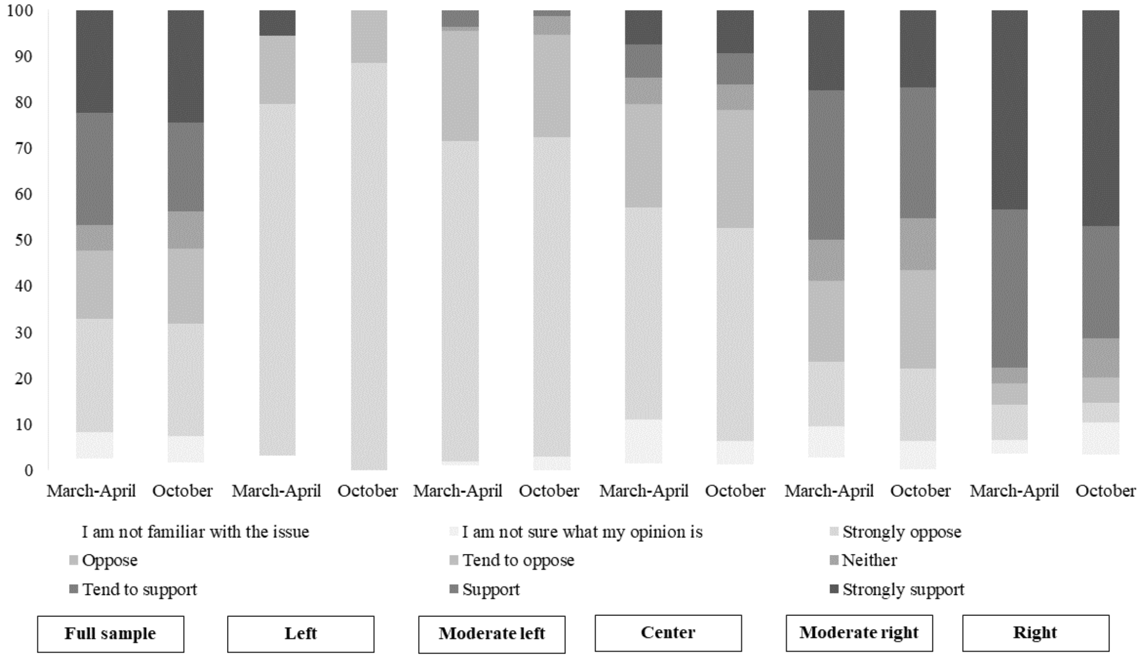

Supplementary Material Figure 1 shows that views on judicial reform were most divided among right-leaning respondents. In March–April 2023, among those who self-identified as "moderate right" (an estimated one-third of the Jewish population in Israel), approximately 50 percent supported the reform, about 32 percent opposed it, and another 16 percent remained neutral. By October, support among the moderate right had declined to approximately 45 percent, while opposition rose to about 37 percent, and around 17 percent remained neutral. In contrast, views in the center and left (including individuals identified as "moderate left") were far more unified in opposition to the reform. The judicial reform debate and accompanying protests thus not only sharpened the divide between the right and left but also fractured the political right, leading many to reconsider their party allegiances.

Based on our data, we estimate that between the November 2022 elections and April 2023, Likud and HaTzionut HaDatit (a religious right-wing party that was the second-largest in the governing coalition) each lost about one-third of their voters (results are not shown but available upon request). This shift in partisan alignment was closely tied to views on the judicial reform: withdrawal was far more common among opponents of the reform—85 percent among former Likud voters and 81 percent among former HaTzionut HaDatit voters—than among its supporters—28 percent among former Likud voters and 30 percent among former HaTzionut HaDatit voters. Our October 2023 survey, conducted after the October 7 attack and the start of the Gaza war, revealed similar trends: those who had identified with either party in March–April 2023 were more likely to withdraw support by October if they opposed the reform (49 and 48 percent) than if they supported it (39 and 32 percent).

Supplementary Material Figure 2. Views on the judicial reform in October 2023, by party voted for in the November 2022 election and party supported in October 2023.

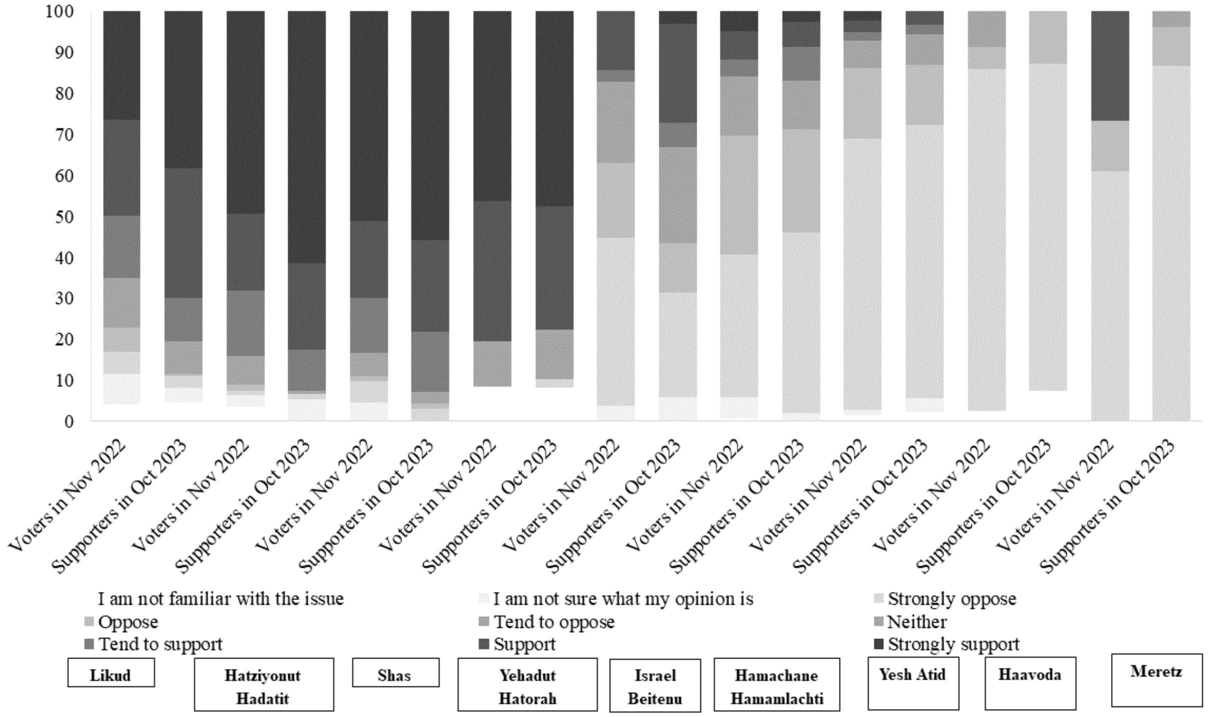

Supplementary Material Figure 2 illustrates the growing alignment between political party support and attitudes toward the proposed judicial reform. For instance, among 2022 Likud voters, approximately 15 percent opposed the reform, and among the roughly 65 percent who supported it, only two-thirds expressed moderate or strong support. By October 2023, however, the overall support had increased to about 80 percent, with nearly 90 percent of supporters expressing moderate or strong support. Meanwhile, opposition among Likud voters dropped to five percent. A similar trend was observed among voters for HaTzionut HaDatit and Shas (an ultra-Orthodox party and the third-largest party in the government). Conversely, support for centrist and left-leaning parties—which was already associated with opposition to the judicial reform in the March–April survey—became even more strongly aligned with anti-reform sentiment in the October survey.

Overall, by October 2023, views on the judicial reform were strongly aligned with partisanship. Among right-wing supporters, 80 percent of Likud supporters and 92 percent of those aligned with HaTzionut HaDatit approved of the reform, with only 5 and 1 percent opposed, respectively. In contrast, among centrist party supporters—HaMachane HaMamlachti and Yesh Atid—opposition was widespread (77 and 84 percent), while support remained low (16 and 5 percent). On the left, support was virtually nonexistent: no respondents affiliated with Avoda or Meretz endorsed the reform, while disapproval stood at 92 and 100 percent, respectively.

Public satisfaction with Prime Minister Netanyahu's leadership followed a similar pattern, as shown in Supplementary Material Figure 3. In both the general sample and among voters from the main right-wing parties, his performance was rated significantly more favorably by supporters of the judicial reform than by its opponents: 61 percent versus 7 percent in the general

sample, 70 versus 20 percent among Likud voters, and 50 versus 30 percent among those aligned with HaTzionut HaDatit.

Supplementary Material Figure 3. View of PM Netanyahu in October 2023 among supporters and opposers of the judicial reform in the general sample and voters of Likud and HaTzionut HaDatit.

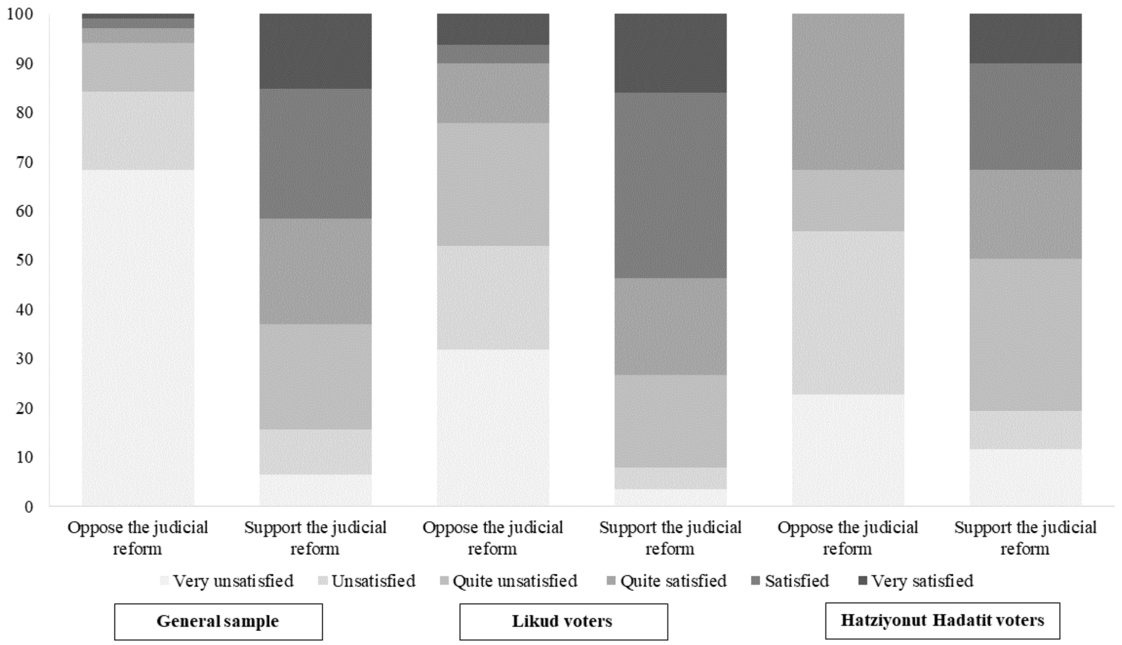

List of References

Feinstein, Yuval. 2025. "Year of Rupture: The Deepening Democratic Divide in Israel in 2023." SocArXiv. September 1. doi:10.31235/osf.io/h28va_v1.
